# Cultural adaptation and validation of the Sinhala version of the Frail Non-disabled tool (FiND)

**DOI:** 10.1186/s12877-024-04749-0

**Published:** 2024-02-14

**Authors:** Shehan Silva, Udayangani Ramadasa, Sarath Lekamwasam

**Affiliations:** 1 Department of Medicine, Faculty of Medical Sciences, University of Sri Jayewardenepura, Nugegoda, Sri Lanka; 2 Department of Medicine, Faculty of Medicine, Sabaragamuwa University of Sri Lanka, Ratnapura, Sri Lanka; 3 Department of Medicine, Faculty of Medicine, University of Ruhuna, Galle, Sri Lanka

**Keywords:** Frailty, Frailty Assessment Tools, FiND Tool, Fried Criteria, PASE Tool, Cross-cultural validation

## Abstract

**Background:**

Frailty, a common geriatric syndrome of vulnerability, is associated with a decline in health and function. The most problematic expression of population ageing is associated with weakness, slowing, decreased energy, lower activity and when severe, unintended weight loss. Frailty is not consciously identified in clinical practice and is not widely studied in Sri Lanka. A validated tool for screening frailty in a busy clinical setting is therefore much needed. This study was done as a part of validating the Sinhala version of the Frail Non-Disabled (S-FiND) tool.

**Methods:**

The FiND tool was translated from English to Sinhala by two translators, blinded to each other. They were combined and translated back to the original language by two separate translators. After verifying the content validity, unambiguity and clarity of items in a focused group discussion, the pre-final version was piloted among 30 volunteers. After assessing the psychometric properties of the pre-final version, the final version was tested among 100 adults older than 65 years from the Colombo South Teaching Hospital. The tool was compared with Fried’s frailty phenotype taken as the gold standard.

**Results:**

Data were analysed for the agreement with the reference standard, the Fried Phenotype. The mean (SD) age of subjects was 73.9 (7.8) years. The overall time taken to fill out the questionnaire was 2 min. The agreement (Kappa) between the S-FiND questionnaire and the Fried phenotype was 0.7 (*P* < 001). The sensitivity and specificity of FiND in detecting frailty were 92% and 74%, respectively. The agreements (Kappa) between the individual items of S-Find: involuntary loss of weight/ more than 4.5 kg over one year, the feeling of effort/ not getting going and level of physical activity, with the Fried phenotype, were 0.28 (*p* = 0.001), 0.06 (*p* = 0.045) and 0.339 (*p* < 0.001). respectively. When subjects were categorized frail and robust based on FiND, frail subjects reported a higher incidence of falls (50%) during the previous 12 months, compared to those robust (13%) (*p* < 0.001 for Chi stat).

**Conclusion:**

The S-FiND is a reliable, valid and well-received tool that can be used in detecting the frailty of non-disabled Sinhala-speaking older adults.

## Background

Frailty leads to an accelerated decline in health and bodily function in old age. It is an expression of homeostatic vulnerability associated with weakness, slowing, decreased energy, lower activity and when severe, unintended weight loss. As a result, people with frailty are vulnerable to procedural complications, falls, institutionalization, disability, and death [1]. 

A consensus group of delegates from international societies created four major key points on frailty and they state that physical frailty is a medical syndrome associated with multiple causes and contributory factors and characterized by diminished strength, endurance, and physiologic function. Further, physical frailty can potentially be prevented or treated with specific modalities, such as exercise, protein-calorie supplementation, vitamin D and avoiding polypharmacy. Simple and rapid screening tests such as the FRAIL scale have been validated and made available enabling physicians to objectively recognize physical frailty [2, 3]. Further, it has been recommended that people older than 70 years and all individuals with unintentional weight loss (> 5%) due to chronic disease should be screened for frailty [2]. 

The Frail Non-Disabled (FiND) tool is a self-completion questionnaire, used as a frailty screening tool in the community setting [4]. Although there are many scales to detect frailty in clinical practice, the majority lack client-driven assessment [5, 6]. Client-driven assessment methods help early identification of diseases or high-risk individuals and are used in other conditions such as osteoporosis and heart failure [7]. The FiND tool, similar to the Fried phenotype has a multidimensional construct of the frailty phenotype [8]. Furthermore, it has been designed to differentiate frailty from disability, two conditions that may overlap but need different management strategies [9–12]. The FiND tool is composed of 5 items which assess physical disability and components of frailty syndrome (Table 1). Physical disability is assessed by 2 questions, inquiring about the ability to walk a 400-meter distance or to climb a flight of stairs. The remaining three questions assess the different components of frailty syndrome: weight loss, exhaustion, and sedentary lifestyle. Patients who present with one or more of the frailty parameters in the absence of mobility disability are defined as those who are frail. Those who have either of the two items of disability irrespective of the components of frailty are considered disabled. Those who do not possess any of the 5 attributes are categorised as robust.

**Table 1:** The Frail Non Disabled (FiND) Tool

Domain	Question	Answer	Score
Disability	A. Have you any difficulties at walking 400 m?	No or some difficulties	0
		A lot of difficulties or unable	1
	B. Have you any difficulties at climbing up a flight of stairs?	No or some difficulties	0
		A lot of difficulties or unable	1
Frailty	C. During the last year, have you involuntarily lost more than 4.5 kg?	No	0
		Yes	1
	D. How often in the last week did you feel that everything you did was an effort or that you could not get going	Rarely or sometimes (twice or less/week)	0
		Often or almost always	0
	E. Which is your level of physical activity?	Regular physical activity (at least 2–4 h/week)	0
		None or mainly sedentary	1

Key:

If questions A + B ≥ 1, the individual is considered as ‘disabled’.

If questions A + B = 0 and C + D + E ≥ 1, the individual is considered as ‘frail’

If A + B + C + D + E = 0, the individual is considered as ‘robust’

Recognizing the importance of early detection and effective management, the British Geriatric Society has recommended regular screening of older people for frailty in all clinical settings and has made such screening an essential component of the comprehensive geriatric assessment (CGA) [13]. Geriatric medicine is an emerging subspecialty in Sri Lanka, hence older adults are managed in medical setups which are busy and not designed to assess age-related medical conditions. Screening for age-related issues is sporadic and depends on the interest of individual physicians or care teams. Further, the poor awareness among doctors about age-related medical issues and the lack of reliable and rapid screening tools hamper the detection and management of frailty in Sri Lanka. Therefore a practical and simple tool to detect frailty is a timely need in Sri Lanka. This study was designed to assess the psychometric properties of the FiND questionnaire translated into Sinhala language.

## Methods

The cross-cultural adaptation of the FiND was performed adhering to the standard guidelines described by Beaton et al. [14]. The original English version was translated into the Sinhala language by two independent health professionals conversant in both languages. One person was informed of the details of the study while the other was not. The two translations were consolidated into one document by the principal investigator in the presence of the two translators. This was done to improve the clarity, ease of comprehension and unambiguity of the items. This version was reverse-translated to English by two different health professionals to determine the comparability with the original version. A group of experts consisting of two physicians and one community physician together with the principal investigator reviewed the translated questionnaire to ensure clarity, face validity, content validity and semantic equivalence.

The pre-final version was piloted among 30 older adults from the study centre to ensure the internal consistency of the questionnaire. The final version (S-FiND) was administered to 100 consented older adults who were not disabled according to the S-FiND questionnaire. Consenting adults aged 65 years or above were recruited from general medical clinics of Colombo South Teaching Hospital. Assuming a power of 80%, Cronbach’s alpha for the null hypothesis of 0.5 and expected Cronbach’s alpha of 0.7, a sample of 90 was required. We recruited 100 older adults who presented to the study settings by purposive sampling method. Data were collected by medical officers who were informed about the study and trained to collect the necessary information.

Informed written consent was obtained from the participants before data collection. This tool was compared against the clinician-detected Fried’s frailty phenotype which was considered the reference standard.

The clinicians who administered Fried’s phenotype, the reference standard, were specialists qualified in internal medicine with a special interest in geriatric medicine and have been in active clinical practice for more than five years.

Fried’s frailty phenotype consists of five criteria: weight loss, exhaustion, low physical activity, slowness and weakness [8, 15]. In the current study, weight loss was defined as an unintentional loss of more than 4.5 kg for 6 months or a body mass index (BMI) of less than 18.5 kg/m^2^ [8, 16]. Exhaustion was considered present when the subject responded as “a little” or ”none of the time” for the Short Form 12-item survey (SF-12) question ‘How much of the time during the past four weeks did you have a lot of energy?’ [8]. A score of less than 73 on the Physical Activity Score of Elderly (PASE) questionnaire was taken as a positive response for low energy expenditure [9]. Slowness was measured as the average of two readings in 6 m, a fast gait speed test and a gait speed of fewer than 0.65 m/s [8]. Weakness was assessed by hand grip strength using a Handheld dynamometer (< 25 kg was considered ‘weak’) [15]. In the Fried questionnaire items are scored in a binary fashion [8, 16]. The composite score of the Fried criteria categories patients into robust and frail (< 3 and 3–5 respectively).

Ethical clearance for the study was obtained from the Ethics Review Committee of the Faculty of Medicine, Sabaragamuwa University of Sri Lanka. The study was performed and adhered to the ethical standards stated in the Declaration of Helsinki [17].

The internal consistency of the FiND questionnaire was determined by Cronbach alpha and total-item correlations. The concurrent validity of S-FiND was tested considering the physician-diagnosed frailty based on the Fried questionnaire, as the reference standard. In addition to the total score, the sensitivity and specificity of individual items of S-FiND were also tested.

## Results

The study subjects were 100 ambulatory older adults, aged 65 years or more, attending medical clinics of a tertiary care centre for more than 6 months. Forty-five of them had diabetes while the prevalence of hypertension, coronary disease, stroke and malignancy were 51%, 30%, 12% and 1%, respectively. The mean (SD) age of participants was 73.9 (7.8) years and 67% were women. The mean (SD) Barthel index was 70.2 (20.4). They had no difficulty understanding the S-FiND questionnaire and were able to complete it in approximately 2 min.

According to the reference standard Fried phenotype 34%, were robust while 66% were frail. The corresponding values based on the S-FiND questionnaire were 30% and 70%. The overall agreement (Kappa) between the S-FiND questionnaire and Fried phenotype was 0.7 *p* < 0.001). Furthermore, compared to the Fried phenotype, the sensitivity and specificity of the total score of S-FiND were 92% and 74%. When the individual items (items 3–5) of S-FiND were compared, specificity showed a variation of 76.5 to 91.2%, while sensitivity remained around 50% (Table 2). The agreement (Kappa) between the individual items of S-FiND and the Fried phenotype is shown in Table 3.

**Table 2:** The sensitivity and specificity of individual items of the S-FiND tool compared with the Fried phenotype

Item Number	Item	Sensitivity	Specificity	Pearson χ^2^	*p* Value
3	During the last year, have you involuntarily lost more than 4.5 kg	48.5%	85.3%	10.984	0.010
4	How often in the last week did you feel that everything you did was an effort or that you could not get going?	56.1%	76.5%	4.010	0.045
5	What is your level of physical activity?	50%	91.2%	16.513	< 0.001

**Table 3:** The agreement (Kappa) between the individual items of S-FiND and the Fried phenotype

Item Number	Item	Kappa Value	*P* Value
3	During the last year, have you involuntarily lost more than 4.5 kg	0.280	0.001
4	How often in the last week did you feel that everything you did was an effort or that you could not get going?	0.061	0.045
5	What is your level of physical activity?	0.339	< 0.001

When the subjects were categorized as either frail or robust based on the S-FiND, they were not different in age and gender distribution. However, a higher incidence of falls during the previous 12 months was observed among those frail compared to robust (50% vs. 13%, *p* = 0.001). Furthermore, compared to those robust, frail subjects had a higher number of current medications (mean 5.5 vs. 3.0, *p* < 0.001) although the Charlson comorbidity index was not significantly different (mean CCI 4.9 vs. 4.1, *p* = 0.15).

## Discussion

We found the S-FiND to have adequate psychometric properties to be used as a screening tool to detect frailty among Sinhala conversant patients. The internal consistency observed is adequate for a community screening tool. According to Cortina, a questionnaire with Cronbach alpha 0.7 or greater is suitable for a screening tool [18].

The FiND tool has been translated and tested among Brazilian Portuguese and the authors have demonstrated its validity in identifying frail old persons sans disability in the community [19]. Similar to our analysis, the authors have used Fried’s phenotype for the comparison. Furthermore, Mirabelli et al. used the FiND to assess frailty among patients with vascular disease [20].

S-FiND demonstrated a higher degree of sensitivity and specificity in detecting patients with frailty without disability and these values are concordant with previous validations. Cesari et al. in the original validation of the FiND tool observed a specificity of 95% (CI 75.1–99.2%) and a sensitivity of 76% (54.9–90.6%). The Brazilian Portuguese version showed 80.5% and 83.3% sensitivity and specificity, respectively.

We found items 3 and 5 to be the main contributors to the overall specificity of the tool. Item 4 compared to item 5, has poor specificity and this may be attributed to the difficulty in understanding the concept of “effort” when translated into the local language. This, however, was not observed in the pilot study performed with 30 participants. The cultural diversity of the perception of health and symptoms is well known and a multitude of culture-related variables and processes are considered to be responsible for this disparity [21]. Despite this limitation, there was a good overall agreement (kappa) between the outputs of S-FiND and FRIED phenotype [22]. 

Our study showed that there was a greater incidence of falls among frail subjects, compared to those robust. Also, those frail were on more medications compared to the robust group. The association between frailty and falls and polypharmacy is well documented. Thakkar et al. and Lu et al. showed a positive association between falls and frailty [23, 24]. while other studies have shown polypharmacy to be associated with frailty [25]. The positive associations between the S-FiND with these variables demonstrate the satisfactory construct validity of the tool.

We found the Sinhala translation of the FiND to be user-friendly and acceptable. Further, they needed minimal assistance in answering the questionnaire. This study was done in an outdoor medical clinic in a tertiary care centre and this can be seen as a limitation of the study. The study participants, however, were ambulatory community-dwelling older adults and we feel that our results can be generalized to other adults in other clinical settings. The validated S-FiND will provide clinicians in Sri Lanka a rapid and reliable method to detect frailty among Sinhala conversant older adults and this can be a positive step in enhancing geriatric services in the country. Although clinicians are aware of the negative impact of frailty, older patients are routinely evaluated for either disability or frailty and this allows clinicians to detect the two distinctive conditions and take appropriate measures in the management of patients. Also, the outcome of this study can provide a platform for future research in this area and we encourage researchers to conduct further studies with larger study samples and diverse clinical settings.

## Conclusions

The validated S-FiND tool has adequate psychometric properties to be used as a simple tool to detect frailty among older adults. It is a rapid user-friendly self-assessment requiring less than 2 min to complete; hence it can be included even in busy clinical setups. We hope that this self-assessment will encourage individuals with health-seeking behaviour to assess their functional capacity and detect frailty early. Furthermore, since the FiND is the only tool that differentiates frailty from disability, it will help detect both categories and initiate appropriate measures to mitigate their physical limitations [26]. 

## Data Availability

The datasets used and/or analysed during the current study are available from the corresponding author upon reasonable request.

## Declarations

### Authors’ information

SS is a senior lecturer in medicine at the University of Sri Jayewardenepura and a board-certified specialist in internal medicine/ geriatrics. UR is a senior lecturer in medicine at Sabaragamuwa University of Sri Lanka and a board-certified specialist in internal medicine. SL is a professor of medicine and a past Dean of the Faculty of Medicine at, the University of Ruhuna. SL was a past president while SS was a past secretary of the Sri Lankan Association of Geriatric Medicine.

## Abbreviations

BMI: Body mass index
CGA: Comprehensive Geriatric Assessment
FiND: Frail Non-Disabled tool
PASE: Physical Activity Score of Elderly
S-FiND: Sinhala version of FiND tool
SF-12: Short Form 12-item survey
